# Self-Reported Compliance with Personal Preventive Measures among Office Workers After Work Resumption during the COVID-19 Outbreak in Pakistan

**DOI:** 10.4269/ajtmh.21-0570

**Published:** 2021-10-04

**Authors:** Pankaj Kumar, Aruba Sohail, Mir Umer Farooq Alam Shah, Maman Khurshid, Farah Yasmin, Muhammad Sohaib Asghar

**Affiliations:** ^1^ Department of Internal Medicine, Dow University of Health Sciences, Karachi, Pakistan;; ^2^ Department of Internal Medicine, Dow University of Health Sciences—Ojha Campus, Karachi, Pakistan

## Abstract

As the lockdowns have been lifted to relieve pressure on the economy, strict adherence to personal preventive measures (PPMs) in offices is essential to control the COVID-19 pandemic. This study investigated self-reported compliance with three PPMs among a sample of office workers in Karachi, Pakistan. A cross-sectional survey with participants were adult office workers who had resumed work in Karachi, Pakistan. All full-time employees aged ≥ 18 years who had resumed work in the offices were invited to complete the survey. Of 487 workers who were invited, 411 (84.4%) completed the survey between March 1 and March 20, 2021. We examined the effects of sociodemographic factors, individual-level factors, interpersonal-level factors, and social-structural–level factors using logistic regression models. Of the total sample, 192 (46.7%) reported always wearing a face mask in the workplace. Self-reported sanitizing of hands (46.0%) was comparable to use of face masks, whereas avoiding crowded places (21.2%) was less common. Perceived effectiveness of individual preventive measures (adjusted odds ratios [AORs] from 1.19 to 1.42; confidence intervals (CIs) 1.04–1.37 to CI 1.18–1.71), perceived effectiveness of governmental preventive measures (AORs from 1.23 to 1.39 CI 1.02–1.47 to CI 1.12–1.72), and number of preventive measures implemented by the office (AORs from 1.20 to 1.26 CI 1.09–1.31 to CI 1.13–1.39) were associated with self-reported compliance with PPMs. Perceived preparedness of medical system in Karachi (AOR 1.44, CI 1.08–1.93) was only associated with self-reported sanitizing of hands. Reduced compliance to PPMs was observed; hence, efforts need to be made to ensure strict adherence to PPMs.

## INTRODUCTION

As of April 28, 2021, 149,368,127 cases of COVID-19 and 3,149,698 deaths from the disease have been reported worldwide.^1^ Pakistan has reported 810,231 confirmed COVID-19 cases and 17,530 deaths.^1^ Initially a nationwide lockdown was imposed by the government to curb the spread of the virus. However, such restrictions adversely affected the country’s economy. Pakistan, being a low- to middle-income country, could not afford extended periods of lockdown. Hence, the government of Pakistan decided to relax the lockdown in phases.^2^ The government released strict guidelines and standard operating procedure documents to ensure safe resumption of work in offices. According to the WHO, indoor locations, especially settings that are enclosed and confined with poor or no ventilation, are riskier than outdoor locations.^3^ Also, people can be in close proximity to each other for long hours in offices. This makes it essential to ensure compliance to personal preventive measures (PPMs) in offices to reduce the risk of contraction.

The WHO released guidelines suggesting the use of face masks, hand hygiene, and physical distancing as PPMs against the virus.^4^ Studies from China^5^ and Australia^6^ reported that a high compliance (80–95%) with PPMs played a pivotal role in curtailing the spread of the virus in these countries. If strict compliance with preventive measures is maintained in offices and other businesses, Pakistan would be able to keep its fragile economy operational amid the pandemic. However, the compliance with PPMs is dependent on many factors. Investigating those factors is important in ensuring an effective infection control. In southern Ethiopia, good knowledge was observed to be a significant factor associated with the participants’ actual practice of the PPMs.^7^ In Lebanon, COVID-19 related news increased knowledge and inculcated fear which ultimately led to increased compliance with ppms.^8^ An international study stated that women were somewhat more likely to engage in these health behaviors than men, but age was unrelated to voluntary compliance behaviors.^9^ Lastly, in Australia, avoidance-related behaviors (avoiding crowds, public transport, and complying with quarantine restrictions) were associated with trust in government/authorities and higher perceived rating of effectiveness of behaviors.^10^ At the social-structural level, implementation of organizational preventive measures during work resumption may differ across offices, which may also affect compliance with personal preventive measures.

To the best of our knowledge, no study has investigated self-reported compliance with personal preventive measures and associated factors among workers who resumed work during the COVID-19 pandemic in Karachi, Pakistan. This study examines the effects of sociodemographic factors, individual-level factors (knowledge and perception), interpersonal-level factors (exposure to COVID-19–specific information through different media), and social-structural–level factors (preventive measures implemented by the offices) on compliance to three personal preventive measures among a sample of office workers in Karachi, Pakistan.

## METHODS

### Study design and sample size.

We conducted a cross-sectional survey of employees who had resumed work in offices during the COVID-19 pandemic in Karachi, Pakistan, between March 1, 2021 and March 20, 2021. The restrictions were eased in phases from May 9, 2020, hence the study was conducted almost a year following the initial lifting of lockdown. We approached eight offices, which included pharmaceutical companies, advertising companies, insurance companies, and banks. Pharmaceutical companies belonged to the health industry. We used OpenEpi: Open Source Epidemiologic Statistics for Public Health to calculate sample size by following single population proportion assumptions of 95% confidence level (CI), expected prevalence of intention to practice PPMs of 50%, and 5% degree of precision.^11^ The sample size calculated using these assumptions was 384. The questionnaire was sent to 487 employees to account for nonrespondents so that the required sample size could be met. Ethical approval was obtained from institutional review board of Dow University Hospital. We received responses from 411 employees, resulting in a positive response rate of 84.4%. Almost half of these responses came from those used in advertising companies (49.6%), followed by bankers (25.2%), pharmaceutical companies (13.3%), and insurance staff (11.9%). Finally, response from 411 office workers was recorded.

### Participants and data collection.

Since the restrictions were relaxed, many offices in Karachi had resumed work. Full-time employees aged ≥ 18 years who had resumed work in offices were invited to complete the survey. Part-time employees and employees working from home were excluded from the study.

A web-based questionnaire was developed using Google forms. The members of the research team visited many offices that had resumed work during the pandemic. After taking permission from the administration, the link to the questionnaire was disseminated among the employees using e-mail. Hardcopies of the questionnaire were also distributed among those employees who did not have access to electronic devices or the internet. Some of the employees were also approached through LinkedIn and other professional networking websites. The link to the questionnaire was sent only after assessing whether the employees fit the inclusion criteria or not. Reminders were also sent after every 3 days to ensure the completion of the survey by the participants. Web-based informed consent was obtained. The participation of the volunteers was entirely voluntary. The survey contained 39 items and required approximately 5 minutes to complete. Google form performed completeness checks before the questionnaire was submitted. Only the corresponding authors had access to the data collected and anonymity of responses was ensured at all stages of study.

### Measures.

#### Design of the questionnaire.

The questionnaire was adapted from the WHO Survey tool and Guidance on COVID-19 and literature related to our study.^12^^,^^13^ The questionnaire was made in both English and the native language Urdu to ensure that all the items were understandable to the participants. The questionnaire was pilot-tested among eight office workers to assess its clarity. These workers did not participate in the actual survey. Based on the participants’ comments, the questionnaire was revised and all the ambiguities were removed.

#### Self-reported compliance with PPMs in the past month.

Participants were asked to report the frequency at which they wore face masks in their offices, and in other public settings apart from their workplaces (public transport) in the past month (response categories: every time, often, sometimes, never). A new variable was created that was termed as “consistent wearing of facemasks in any public place.” This composite variable referred to the participants who reported always using a face mask both in the workplace and other public settings such as public transport. The participants were also asked about the type of face mask they used and whether they reused their face masks. The participants also reported the frequency at which they sanitized their hands using soaps, liquid soaps, and alcohol-based sanitizers after returning from public places or touching public installations, and whether they avoided crowded places or not (response categories: every time, often, sometimes, never).

#### Background characteristics.

Participants were asked to report sociodemographic characteristics such as gender, age, marital status, highest education level attained, and designation in the office.

#### Individual-level variables.

To determine the participants’ knowledge related to transmission routes of COVID-19, a composite indicator variable was constructed by counting the number of correct responses to five knowledge items related to COVID-19 transmission routes (ranging from 0 to 5). To assess their perceptions related to COVID-19, three scales were constructed for this study: 1) the four-item Perceived Severity Scale, 2) the four-item Perceived Effectiveness of Individual Preventive Measures Scale, and 3) the 2-item Perceived Effectiveness of Governmental Preventive Measures Scale. The response categories for these scales were 1 = disagree/ineffective, 2 = neutral, and 3 = agree/effective. The Cronbach alpha values of all three scales were > 0.7, and single factors were identified by exploratory factor analysis that cumulatively explained 61.6% of the total variance. A single item was used to measure the perceived preparedness of the medical system of Karachi with the same response categories as the scales. In addition, a single item was used to measure the participants’ perceived risk of contracting COVID-19 in the next 3 months (response categories: 1 = low, 2 = moderate, 3 = high), and another item measured the perceived effectiveness of preventive measures implemented by the offices (response categories: 1 = very ineffective, 2 = ineffective, 3 = neutral, 4 = effective, 5 = very effective).

#### Interpersonal-level variables.

A single item was used to assess the daily average time (hours) of exposure to COVID-19–specific information through TV, newspaper, social media, and interpersonal communication. The response categories for the aforementioned items were 1 = almost none, 2 = less than 1 hour, 3 = 1–2 hours, 4 = 3–4 hours, and 5 ≥ 4 hours.

#### Social-structural-level variables.

The study participants were asked to report whether their offices had implemented the nine preventive measures to allow safe resumption of work in the offices. A composite indicator variable was also created by counting the number of preventive measures implemented by the office (ranging from 0 to 9).

### Statistical analysis.

The dependent variables were self-reported consistent wearing of a face mask in public spaces, sanitizing hands every time after returning from public spaces or touching public installations, and always avoiding crowded places. Logistic regression models were chosen to analyze the factors associated with dependent variables. Univariate logistic regression was used to assess the significance of the association between each of the background characteristics and the dependent variables. Statistical Package for the Social Sciences (SPSS) version 23.0 for Windows (IBM Corporation) was used for data analysis and *P *≤ 0.05 was considered statistically significant.

## RESULTS

### Background characteristics.

We approached 487 office employees who had resumed work during the pandemic to fill the survey, and 411 of them completed the survey, for an overall response rate of 84.4%. Nearly three-quarters (72.3%), of the employees were aged > 30, married (74.2%) and appointed at high-ranking positions in their offices (72.5%). The majority were men (86.1%) and had completed tertiary education (91.7%) (Table 1).

**Table 1:** Background characteristics of participants (*N* = 411)

Characteristics	Value (%)
Age (years)	
18–25	47 (11.4)
26–30	67 (16.3)
31–40	152 (37.0)
> 40	145 (35.3)
Gender	
Male	354 (86.1)
Female	57 (13.9)
Relationship status	
Married	305 (74.2)
Unmarried	106 (25.8)
Highest level of education attained	
Postgraduate	234 (56.9)
College/university	143 (34.8)
Intermediate	23 (5.6)
Matriculation	11 (2.7)
Occupational position	
Chief operating officer/director/ general manager (heads)	125 (30.4)
Managers	173 (42.1)
Officers	73 (17.8)
Cleaning staff/receptionist	23 (5.6)
Others	17 (4.1)

### Self-reported compliance with PPMs in the past month.

In the past month, 192 participants (46.7%) reported always wearing facemasks in the workplace, and 228 participants (55.5%) reported always wearing a face mask in other public settings. One hundred twenty-seven (30.9%) participants reported “consistently wearing a face mask in any public place.” Surgical masks were the choice of 77.9% of the participants, and 64.2% of the participants reused their face masks. Self-reported sanitizing of hands (46.0%) was comparable to compliance with the use of face masks, whereas avoiding crowded places (21.2%). Tables 2 and 3 show the responses to the survey items measuring the individual and interpersonal variables, and Table 4 reports the responses to social-structural–level variables.

**Table 2:** Self-reported compliance with personal protective measures related to COVID-19 (*N* = 411)

Measures and responses	*N* = 411 (%)
Frequency of face mask wearing in the workplace	
Every time	192 (46.7)
Often	148 (36.0)
Sometimes	67 (16.3)
Never	4 (1.0)
Frequency of facemask wearing in public places except workplace (e.g., public transportation)	
Every time	228 (55.5)
Often	122 (29.7)
Sometimes	53 (12.9)
Never	8 (1.9)
Consistent wearing of face mask in any public place	
Yes	127 (30.9)
No	284 (69.1)
Type of face mask worn	
Surgical mask	320 (77.9)
Cloth mask	49 (11.9)
N-95 mask	42 (10.2)
Reuse of face mask	
No	147 (35.8)
Yes	264 (64.2)
Frequency of hand sanitization (using soap, liquid soap or alcohol-based sanitizer) after returning from public spaces or touching public installations	
Every time	189 (46.0)
Often	155 (37.7)
Sometimes	64 (15.6)
Never	3 (7.0)
Avoiding crowded places	
Always	87 (21.2)
Often	228 (55.5)
Sometimes	87 (21.2)
Never	9 (2.2)

**Table 3:** Responses to survey items measuring individual-level variables (N = 411)

Variable	Value
Daily average time to COVID-19–related information through different official media channels (answered > 1 hour), *n* (%)	195 (47.4)
Daily average time to COVID-19–related information through different official media channels, mean (SD)	2.17 (2.5)
Knowledge about route of transmission of COVID-19 (answered yes), n (%)	
Contact with droplets	336 (81.8)
Touching contaminated objects	344 (83.7)
Direct contact with wildlife	53 (12.9)
Contact with feces	66 (16.1)
Contact with asymptomatic patients	247 (60.1)
Correct responses to COVID-19 transmission route questions	
Number of correct responses, mean (SD)	3.42 (1.1)
0 correct response, n (%)	4 (1.0)
1 correct response, n (%)	17 (4.1)
2 correct responses, n (%)	60 (14.6)
3 correct responses, n (%)	119 (29.0)
4 correct responses, n (%)	144 (35.0)
5 correct responses, n (%)	67 (16.3)
Perceptions related to COVID-19	
Perceived risk of contracting COVID-19 (answered high), n (%)	48 (11.7)
Perceived risk of contracting COVID-19, mean (SD)	2.3 (0.7)
Perceived consequences of COVID-19 (answered agree), n (%)	
Permanent bodily damage to infected people	131 (31.9)
High mortality rate of infected people	121 (29.4)
Lack of effective treatment	247 (60.1)
Lack of effective vaccines for prevention	306 (74.5)
Perceived Severity Scale score, mean (SD)	9.0 (1.7)
Perceived effectiveness of individual-level preventive measures (answered effective), N (%)	
Wearing face masks	371 (90.3)
Sanitizing hands frequently	378 (92.0)
Household disinfection	285 (69.3)
Avoiding gatherings	350 (85.2)
Perceived effectiveness of Individual Preventive Measures Scale score, mean (SD)	11.1 (1.6)
Perceived effectiveness of preventive measures taken by the office, (answered effective or very effective), N (%)	245 (59.6)
Perceived effectiveness of preventive measures taken by the office, mean score (SD)	3.4 (1.2)
Perceived effectiveness of governmental preventive measures (answered effective), N (%)	
Closure of public spaces (e.g., restaurants, theatres etc.)	239 (58.2)
Smart lockdowns	282 (68.6)
Perceived effectiveness of governmental preventive measures scale score, mean (SD)	5.0 (1.1)
Perceived organizational preparedness for COVID-19 outbreak after work resumption (answered agree), m (%)	
The medical system in Karachi is well prepared for a COVID-19 outbreak after work resumption	54 (13.1)
Perceived preparedness scale score, mean (SD)	1.5 (0.7)

**Table 4:** Response to items measuring social-structural level variables (*N* = 411)

Preventive measures implemented by the office	Office workers (answered yes) n (%)
Taking body temperature and requiring hand sanitation for all employees entering the workplace	335 (81.5)
Providing face masks to all employees	312 (75.9)
Requiring employees to wear face masks in the workplace	335 (81.5)
Frequent workplace disinfection	277 (67.4)
Practicing social distancing in workplace	240 (58.4)
Random testing of employees	163 (39.7)
Employees are called to the office on alternate days	216 (52.6)
Leaves granted to COVID-19–infected individuals	367 (89.3)
Mandatory 14-day quarantine for employees physically interacting with COVID-19 positive people	323 (78.6)

### Factors associated with self-reported compliance with PPMs in the past month.

In the univariate logistic regression analysis, age, gender, education level, and job position were significantly associated with self-reported compliance with one or more PPMs. The odds of wearing a face mask consistently in public place were 2.3 times higher in females (odds ratio [OR] 2.29, 95% confidence interval [CI] 1.29–4.04; *P* = 0.004) compared with males. The odds of sanitizing hands were significantly higher for those who received tertiary education (college/university and postgraduate) compared with those who have only done intermediate or matriculation. People aged 26 to 40 years had a significantly higher odds of compliance toward consistent hand sanitizing in the workplace. Lower job ranks were significantly associated with reduced odds of avoiding crowds compared with heads of the organizations as shown in Table 5.

**Table 5:** Associations between background characteristics and self-reported compliance with different personal preventive measures

Characteristic	Wearing a face mask consistently in public place		Sanitizing hands every time after returning from public space or touching installations		Always avoiding crowded places	
	OR (95% CI)	*P* value	OR (95% CI)	*P* value	OR (95% CI)	*P* value
Age (years)						
18–25	Reference	N/A	Reference	N/A	Reference	N/A
26–30	0.93 (0.41–2.12)	0.87	2.43 (1.11–5.34)	**0.03**	0.83 (0.31–2.19)	0.71
31–40	1.06 (0.52–2.15)	0.88	2.24 (1.11–4.51)	**0.03**	1.26 (0.56–2.86)	0.58
> 40	1.13 (0.55–2.31)	0.74	2.03 (1.00–4.10)	**0.05**	1.20 (0.52–2.73)	0.67
Gender						
Male	Reference	N/A	Reference	N/A	Reference	N/A
Female	2.29 (1.29–4.04)	**0.004**	0.98 (0.56–1.72)	0.95	0.88 (0.43–1.77)	0.71
Relationship status						
Unmarried	Reference	N/A	Reference	N/A	Reference	N/A
Married	0.83 (0.52–1.32)	0.43	1.15 (0.74–1.80)	0.54	1.03 (0.60–1.78)	0.90
Highest level of education attained						
Matriculation	Reference	N/A	Reference	N/A	Reference	N/A
Intermediate	0.25 (0.04–1.81)	0.17	4.38 (0.47–41.07)	0.20	0.95 (0.15–6.17)	0.96
College/university	1.07 (0.27–4.24)	0.92	11.03 (1.38–88.43)	**0.02**	1.15 (0.24–5.59)	0.87
Postgraduate	1.41 (0.37–5.47)	0.62	8.28 (1.04–65.74)	**0.046**	1.29 (0.27–6.14)	0.75
Job position						
Heads	Reference	N/A	Reference	N/A	Reference	N/A
Managers	0.97 (0.59–1.60)	0.92	1.09 (0.69–1.73)	0.71	0.45 (0.26–0.77)	**0.004**
Officers	1.22 (0.66–2.25)	0.52	0.90 (0.50–1.61)	0.73	0.42 (0.20–0.86)	**0.02**
Cleaning staff/ receptionist	0.33 (0.09–1.18)	0.09	0.32 (0.11–0.92)	**0.03**	0.20 (0.05–0.91)	**0.04**
Others	1.20 (0.42–3.49)	0.73	1.65 (0.59–4.61)	0.34	0.46 (0.12–1.68)	0.24

CI = confidence interval; N/A = not applicable; OR = odds ratio. All bold *P*-values are statistically significant (< 0.05).

After adjusting for these significant background characteristics; perceived effectiveness of individual preventive measures, governmental preventive measures, and number of preventive measures implemented by the office were significantly associated with consistent usage of facemask in any public place, and sanitizing hands every time after returning from public places with the exception of avoiding crowds. Perceived preparedness of medical system in Karachi (adjusted OR 1.44, 95% CI: 1.08–1.93; *P* = 0.01) was only associated with self-reported sanitizing of hands but not with other personal preventive measures as shown in Table 6.

**Table 6:** Factors associated with self-reported compliance with different personal preventive measures

Factor	Consistent wearing of face mask in any public place		Sanitizing hands every time after returning from public spaces or touching installations		Always avoiding crowded places	
	AOR (95% CI)	*P* value	AOR (95% CI)	*P* value	AOR (95% CI)	*P* value
Individual-level variables						
Exposure to COVID-19 related information	1.02 (0.87–1.19)	0.85	1.02 (0.88–1.19)	0.75	1.05 (0.88–1.26)	0.59
Knowledge and perception						
Knowledge about transmission routes of COVID-19	1.13 (0.93–1.37)	0.21	1.01 (0.84–1.22)	0.91	0.97 (0.78–1.22)	0.81
Perceived risk of contracting COVID-19	1.06 (0.77–1.45)	0.72	1.03 (0.77–1.40)	0.83	0.99 (0.69–1.43)	0.96
Perceived severity of COVID-19	1.01 (0.89–1.14)	0.89	1.11 (0.99–1.25)	0.066	1.09 (0.95–1.25)	0.22
Perceived effectiveness of individual preventive measure	1.42 (1.18–1.71)	< 0.001	1.19 (1.04–1.37)	0.01	1.06 (0.90–1.24)	0.52
Perceived effectiveness of preventive measures taken by office	1.14 (0.96–1.36)	0.15	1.16 (0.98–1.36)	0.08	1.07 (0.88–1.31)	0.50
Perceived effectiveness of governmental preventive measures	1.39 (1.12–1.72)	0.002	1.23 (1.02–1.47)	0.03	1.10 (0.88–1.37)	0.41
Perceived preparedness of medical system in Karachi	1.09 (0.82–1.46)	0.56	1.44 (1.08–1.93)	0.01	1.14 (0.82–1.59)	0.43
Social–structural level variable						
Number of preventive measures implemented by the office	1.26 (1.13–1.39)	< 0.001	1.20 (1.09–1.31)	< 0.001	1.08 (0.97–1.20)	0.16

CI = confidence interval; N/A = not applicable; AOR = adjusted odds ratio.

## DISCUSSION

On March 11, 2020, that COVID-19 was declared a pandemic by the WHO.^14^ Lockdowns and restrictions were imposed worldwide to curtail the spread of the virus. Even after almost a year, on February 25, 2021, 430,167 confirmed cases and 10,714 deaths were reported worldwide,^15^ although many countries had started rolling out vaccines. Because countries, especially low- to middle-income countries, cannot afford extended periods of lockdown due to detrimental effects on the economy; compliance to personal preventive measures is necessary in workplaces and public places to control the pandemic. However, in our study less than one-third (30.9%) of office workers reported consistent use of facemasks in any public place. Only 46% and 21.2% of the office workers reported that they sanitized their hands every time and always avoided crowds, respectively. These findings are troublesome because Pakistan is still reporting new confirmed cases and deaths. For example, on February 24, 2021, alone, Pakistan reported 1,361 confirmed cases and 64 deaths from COVID-19 infection.^16^ The vaccines are currently being dispensed among healthcare workers and senior citizens. Hence, it would require a considerable amount of time before the vaccine is accessible to majority of the population and its acceptance increases. Until then, it is important to maintain strict compliance to PPMs.

Pakistan has reported fewer COVID-19 cases and deaths compared with many other countries. As of February 21, 2021, the United States had reported 27,702,074 cases and 491,894 deaths; Iran had reported 1,566,081 cases and 59,409 deaths; and the United Kingdom had reported 4,105,679 cases and 120,365 deaths; in contrast, Pakistan had reported 569,846 cases and 12,563 deaths.^1^ The lower COVID-19 burden in Pakistan could be the reason for not maintaining high compliance to PPMs. This deduction could also be supported by the finding that only 48 (11.7%) employees considered themselves to be at a high risk of contracting the virus. Moreover, the fatalistic belief that if one is destined to contract COVID-19, it becomes unavoidable irrespective of the preventive measures taken also contributes to reduced compliance.

This study highlights issues that should be addressed through targeted interventions. Despite sufficient availability of face masks, ∼64.2% of the participants responded to have reused their facemasks. Efforts should be put in raising awareness regarding the proper handling and disposal of facemasks according to the guidelines set by the WHO.^17^ The reuse of surgical masks should be discouraged, and frequent washing of fabric masks should be encouraged.

According to the findings of the study, interventions should be designed according to the needs of various groups. Male workers were less likely to wear a face mask consistently in their offices compared with female workers. Decreased compliance of males to PPMs has also been reported in other studies.^13^^,^^18^^,^^19^ Efforts should be made to determine factors associated with decreased compliance by males, and they should be educated accordingly. Because the odds of sanitizing hands were higher for those who received tertiary education (college/university and postgraduate) compared with those who have only done intermediate or matriculation, more attention should be given to individuals with lower education levels. Health promoting messages should be designed according to the literacy rate of the employees so that they could be easily interpreted by them. Also, heads of organizations reported greater odds of avoiding crowds compared with those appointed at lower designations. This could be because higher ranks in the organizational hierarchy of an office come with added responsibility. To be role models to their subordinates, they displayed greater compliance to PPMs. Lower odds of sanitizing hands for the age group 18 to 25 could be due to the observation that the rate of hospitalization and death due to COVID-19 is lower for the age-group 18 to 29 compared with ages ≥ 30.^20^

The government of Pakistan issued guidelines regarding the mitigation of spread of person-to-person transmission of COVID-19 to allow safe resumption of work in offices.^21^ At the social-structural level, the preventive measures implemented by the offices played important roles in COVID-19 prevention, as the number of preventive measures implemented by the offices were associated with compliance with two of the personal preventive measures. Some of these measures directly increase access to face masks, such as provision of facemasks (75.9%) and making them mandatory (81.5%). Majority of employees (81.5%) have also reported that measuring body temperature, and requiring hand sanitation upon entering the workplace is a mandatory practice in their workplace. These measures can directly increase compliance to PPMs. However, only 58.4% of the respondents reported that social distancing of 6 feet is practiced in their offices. This finding is troublesome as physical distancing has been strongly advocated by the WHO to control the spread of the virus.^4^ Hence, interventions such as spacing of desks should be made to maintain social distancing. Only 52.6% of the employees have reported that they are called on alternate days by their offices even though implementation of this measure could allow for physical distancing and limit interaction of workers in the workplace.

Perceived effectiveness of individual and governmental preventive measures had a strong influence on compliance with PPMs. The importance of believing that preventive measures would be effective has been highlighted in other studies as well.^9^^,^^10^ Thus, it shows that the campaigns have been effective in raising awareness among people regarding the efficacy of the PPMs in preventing COVID-19. Hence more efforts should be directed toward such campaigns.

This study has some limitations. First, the data were self-reported, and authentication was not feasible. Recall bias might have occurred. Participants may have also overreported their compliance with PPMs due to social desirability. Second, some behavioral factors that may influence personal preventive behaviors during the COVID-19 pandemic were not considered in this study, such as comfort of adopting these preventive measures or personal loss of a loved one due to the pandemic. Third, the study cannot be generalized to all the offices in Karachi because the sample size was limited. Also, the resource constraints of the offices in implementation of the measures was not considered. Lastly, compliance with PPMs despite having contracted COVID-19 has not been investigated in our study.

## CONCLUSION

Gaps were observed in the implementation of all three personal preventive measures. Hence, efforts need to be made to ensure adherence to the guidelines advocated by the WHO for effective infection control in the workplace. Rigorous awareness campaigns need to be carried out at governmental and organizational level. The organization should ensure strict implementation of personal preventive measures by the employees. Factors that affect compliance should be investigated thoroughly. Through this, effective and targeted interventions can be devised at both the government and office levels, thus increasing compliance. Health messages should be designed according to the literacy rate of the employees so that it can be understood by majority of the population.
